# Present Status of Pneumococcus Infections

**Published:** 1916-04

**Authors:** W. W. Young

**Affiliations:** Atlanta, Ga.


// Journal-Record of Medicine

Successor to Atlanta Medical and Surgical Journal, Established 1855
and Southern Medical Record, Established 1870
OWNED BY THE ATLANTA MEDICAL JOURNAL COMPANY
PUBLISHED MONTHLY

*Official Organ Fulton County Medical Society, State Examing Board, Atlanta, Birmingham and Atlantic Railroad Surgeon's Association, Chattahoochee Valley Medical and Surgical Association, Etc.*

RICHARD R. DALY, M. D., Editor
BERNARD WOLFF, M. D., Supervising Editor
A. W. STIRLING, M. D., C. M., D. P. H.; J. S. HURT, B. Ph., M.D.
GEO. M. NILES, M. D., W. J. LOVE, M. D., (Ala.); Associate Editors
E. W. ALLEN, Business Manager
COLLABORATORS
W. F. WESTMORELAND, M. D., General Surgery
F. W. McRAE, M. D., Abdominal Surgery
ALLEN H. BUNCE, Medical Societies
E. B. BLOCK, M. D., Diseases of the Nervous System
MICHAEL HOKE, M. D., Orthopedic Surgery
CYRUS W. STRICKLER, M. D., Legal Medicine and Medical Legislation
E. C. DAVIS, A. B., M. D., Obstetrics
E. G. JONES, A. B., M. D., Gynecology
ALLEN H. BUNCE, M. D., Pathology and Bacteriology
R. T. DORSEY, JR., B. S., M. D., Medicine
L. M. GAINES, A. B., M. D., Internal Medicine
GEO. C. MIZELL, M. D., Diseases of the Stomach and Intestines
L. B. CLARKE, M. D., Pediatrics
EDGAR PAULLIN, M. D., Opsonic Medicine
THEODORE TOEPEL, M. D., Mechano Therapy
R. R. DALY, M. D., Diseases of the Eye, Ear, Nose and Throat
E. G. BALLENGER, M. D., Diseases of the Genito-Urinary Organs

VOL. LXIII. ATLANTA, GA., APRIL, 1916. NO. 1

### PRESENT STATUS OF PNEUMOCOCCUS INFECTIONS.

BY W. W. YOUNG, A.B., M.D., ATLANTA, GA.

The conditions in pneumonia at present are different only in degree from those found in typhoid fever. Formerly all cases running the clinical course of typhoid fever were thought to be incidental. Now it is known that in addition to affection with the typical B. typhosus, we find infections with the Paratyphoid Bacillus A & B. It is true that these organisms differ slightly in cultural characteristics, it being through their behaviour toward the sugars that they were separated into groups. On the other hand, types of Pneumococcus do not differ in cultural reactions but in their biological characteristics.

Most of the cases of acute Lobar Pneumonia appear to be due to the Diplococcus Pneumonia. But work shows that all of these cases due to the Pneumococcus are not identical. In 1905 Schott Muller described in some cases of acute pneumonia a Streptococcus, which always had a capsule and which in the body gave rise to a sticky, tenacious exudate. This organism proved to belong with the Pneumococcus group and was called the Pneumococcus Mucosus. These cases were all extremely severe and showed very high mortality. In 1911 Neufeld and Handel showed that immune serum produced by some races of Pneumococci was not effective against all. Three years ago Dochez and Gillespie, at the Rockefeller Institute, began the study on the various races of Pneumococci by work on the immunity reactions. It was found that groups could be formed whose immune serum caused specific agglutination of all the organisms belonging to that one particular group. The organisms were arbitrarily divided into groups I, II and III, which is the organism Schott Muller called the Pneumococcus Mucosus, and group IV, a group of organisms having no common immunological characteristics.

These organisms show varying proportions of infectivity: Number 1, 38% ; Number II, 30% ; Number III, 11% ; Number IV 21%. So we see that by far the larger number of cases belong to groups I and II, and the significance of this we shall see later. We studied these organisms at the P. B. Brigham Hospital in Boston, in 1913-1914 and confirmed the findings of the Rockefeller Institute. The groups also vary in clinical manifestations and severity, the mortality in the various groups being as follows: Number I, 25% ; Number II, 36% ; Number III, 17% ; Number IV, 6%. So we see that in number III we have very few cases with a very high mortality, and in number IV the mortality is exceedingly low.

The question of epidemiology then presents. We know that a certain percentage of individuals harbor a Pneumococcus in the mouth. The importance of this depends much upon the type of organism and its consequent virulence. Careful study by Dochez and Avery showed that the Pneumococcus found in the mouth of normal individuals not having been in contact with pneumonia patients, belonged to group IV. Study of the mouths of individuals who had been in contact with pneumonia patients showed that they harbored organisms belonging to the more virulent groups. And finally, patients recovering from pneumonia were found to harbor the organism in the mouth for some time after recovery.

Now, it has been shown that, unlike some other organisms, which will infect no matter what the state of the recipient, infection with the Pneumococcus takes place only when the resistance of the recipient is considerably lowered. However, the different groups of Pneumococci have very different degrees of parasitism. So, individuals harboring the more virulent types should be considered carriers. It seems plausible, in view of these considerations, that a forward step in prophylaxis will be the eradication of all carriers of the more virulent types of the Pneumococcus.

Next, it would be interesting to ascertain just what is the noxious agent produced by the pneumococcus which causes the disease. We know that the organisms of Diphtheria and Tetanus grow locally and produce their pathological effects by a poison which they throw off in their metabolic processes. The efforts to find such a toxin in the bodies of animals infected by the Pneumococcus have been fruitless.

If Pneumococci are treated with immune body and complement, if they be placed in salt solution at 37 degrees C. for from 12-20 hours, or treated with sodium chlorate, or simply ground up in the cold and dissolved in salt solution, a solution is obtained which is toxic for guinea pigs when injected intravenously. The symptoms produced resemble those seen in anaphylactic reactions and the toxin is hence called, anaphylatoxin. It seems evident that this toxin is contained in the bacteria and is not a product of their metabolism. These are endotoxins.

There is no evidence at present that this toxin is of any significance so far as the intoxication in Lobar Pneumonia is concerned. On the other hand, there is considerable evidence that it is not through the death but through the life processes that the symptoms of Pneumonia are produced. The injection of dead Pneumococci into experimental animals, except in enormous numbers, has very little effect, whereas the multiplication of living organisms elicits profound symptoms.

There is one phenomenon observed with growing, living Pneumococci which may prove of considerable value. Either in vitro or in vivo the Pneumococcus, growing, will produce metabomoglobin from oxyhemoglobin. Reasoning by analogy from chemical processes in vitro, we must conclude that the bacteria produces some ferment or catalytic agent which, in the neighborhood of the organisms and under differing conditions of oxygenation, produces methemoglobin. This is specific for the Pneumococcus and is brought about by mere contact with the corpuscles. This is interesting because it suggests a possible explanation of the toxic action of the Pneumococcus on the body. If the Pneumococcus can cause a change in this way in the content of the red blood cell, it is conceivable that it may cause other changes in other tissue cells by mere contact and growth. This is merely a surmise based on analogy. However, we have come to feel that the war is to be waged against the organism and not against a specific toxin.

Having gotten more clearly in mind the disease process, the next step is to attempt to combat it. We cannot neglect the importance of heightening and stimulating in every way the individual's resistance. However, something specific is the ideal we seek. With the growing tendency to seek for rational chemo-therapy Morgenroth experimented with various products of quinine. He finally discovered one which he called ethylhydrocuprein, which exerted protective and curative properties in mice infected with Pneumococcus. This chemical attacked and killed the organism in vitro. It was found, however, that this drug was very toxic to man and its practical value is doubtful.

By far the most justifiable method of treatment, in view of our experimental knowledge, is by means of specific sera. The efficiency of Diphtheria and Tetanus antitoxins in neutralizing the specific toxins are the best examples, and this is the reason for trying to learn if there are any toxins elaborated by the Pneumococcus. The attempt to obtain an immune serum by the injection of anaphylatoxin yielded results of very little practical value, inasmuch as such a serum possesses very little protective or curative properties.

By far the most effective serum obtained is that produced by the injection of living organisms. Such an immune serum has been known and used since 1891 but was practically abandoned because of lack of rational results.

It is evident, in the light of the more recent work, why immune sera were found unsatisfactory. With the discovery of varied strains or races of Pneumococci we know that each strain has its own specific serum. So it becomes necessary to produce sera against each strain. We must have some means of quick determination of the specific organism and we must have a method of standardization so that we may be able to determine rational doses of sera.

Sera of high protective power have been produced against types I and II by the immunization of horses with properly spaced doses of living organisms. It is impossible to produce a serum of any protective power against type III. Type IV is so heterogeneous as to render the production of a serum in their case impossible. The mortality of this latter group is so low and the number of cases belonging to group III so small, that these limitations do not detract from the practical value of sera. For, as we have seen, sera may be obtained protective against groups I and II.

The first step is to determine the type of organism infecting the individual. The patient is turned on the affected side and the sputum coughed up is obtained. This is washed in sterile salt solution to wash off the mouth organisms on the surface. A small amount of this sputum is then injected into the peritoneal cavity of a mouse. In from 6-8 hours the mouse either dies or is killed and the peritoneal cavity opened. The exudate obtained contains a pure culture of the offending organism. This is put into sterile salt solution and centrifuged, the organism being thrown to the top. These are decanted and suspended in salt solution. Equal parts of sera I and II in separate test tubes and organism suspension are then mixed and incubated at body temperature for half an hour. If the organism is of either type, I or II, the specific serum will cause an agglutination of the organism, and we have thus identified the type of infection.

In order to standardize these sera mice are used. They are divided into two series, in one of which doses of fresh bouillon cultures of decreasing size from 1 c. c. to 0.000001 c. c. are injected. In the second series of mice similar doses of the culture are injected but in each case this is mixed with 0.2 c. c. of immune serum. This dose of serum has been chosen after many experiments have demonstrated it to be the most suitable. A serum which in doses of 0.2 c. c. will protect a mouse of 15-20 gm. against 0.1 c. c. of culture, of which 0.000001 c. c. alone will kill, has been found always to act in this way. It has been possible to produce an immune horse serum of such a type against organisms of type I. To produce so effective a serum against type II has been found more difficult. Type II serum in doses of 0.2 c. c. usually protects a horse against only 0.01 c. c. of culture and no more. The relative protective power of the body toward the different types varies, that against group IV being greatest; then types I and II, and finally against III the body cannot produce protective bodies.

Large doses of these sera should be used and should be given early in the disease. Usually 80-90 c. c. of serum is given intravenously and is repeated in twelve hours. This is continued as long as the patient's condition seems to make it advisable. The mortality has been materially reduced and the course and the severity of the disease decreased by the use of these sera. It has been found that the immune bodies are contained in a part of the globulin fraction of the protein. So in the future it will be possible to avoid, to a great extent, the occurrence of symptoms of serum sickness by concentration of the serum and immune bodies.

We have thus found out the ruling principles. This must be expanded and elucidated by further work and research. But this is going to be a most fruitful field and its practical value is already evident and cannot but appeal to everyone.

605 Ponce de Leon Ave., Atlanta, Ga.

